# Analysis of novel human papillomavirus type 16 late mRNAs in differentiated W12 cervical epithelial cells

**DOI:** 10.1016/j.virol.2006.10.012

**Published:** 2007-03-30

**Authors:** Steven G. Milligan, Thanaporn Veerapraditsin, Boolang Ahamet, Sarah Mole, Sheila V. Graham

**Affiliations:** Rm 2/14 GBRC, IBLS Division of Infection and Immunity, University of Glasgow, 120 University Place, Glasgow, G12 8TA, Scotland, UK

**Keywords:** HPV, human papillomavirus, RACE, rapid amplification of cDNA ends, mRNA, messenger RNA, LCR, long control region, 3′UTR, 3′ untranslated region, SD, splice donor, SA, splice acceptor, Human papillomavirus type 16, Regulation of gene expression, Transcript mapping, Promoter mapping, Putative novel proteins

## Abstract

The life cycle of human papillomavirus type 16 (HPV16) is intimately linked to differentiation of the epithelium it infects, and late events in the life cycle are restricted to the suprabasal layers. Here we have used 5′RACE of polyadenylated RNA isolated from differentiated W12 cells (cervical epithelial cells containing episomal copies of the HPV16 genome) that express virus late proteins to map virus late mRNAs. Thirteen different transcripts were identified. Extensive alternative splicing and use of two late polyadenylation sites were noted. A novel promoter located in the long control region was detected as well as P_97_ and P_late_. Promoters in the E4 and E5 open reading frames were active yielding transcripts where L1 or L2 respectively are the first open reading frames. Finally, mRNAs that could encode novel proteins E6*^*E7, E6*^E4, E1^*E4 and E1^E2C (putative repressor E2) were identified, indicating that HPV16 may encode more late proteins than previously accepted.

## Introduction

Human papillomaviruses (HPVs) infect cutaneous and mucosal epithelia causing benign lesion, or warts, that can sometimes progress to malignancy (Howley, 1996). A subset of mucosal HPV types infects anogenital epithelia. Infection with “low risk” HPV types 6 and 11 causes genital warts. However, some other anogenital types (e.g. types 16, 18, 31) are described as “high risk” due to the fact that persistent infections with these cause HPV-associated lesions that can progress to cancer. Most notably, HPV16, the most common high risk anogenital type, is associated with 60% of cervical tumours (Walboomers et al., 1999).

HPV16 has a 7.9 kb circular double stranded DNA genome that exists in the nucleus of the infected epithelial cell episomally. Episomal maintenance of the genome is essential for completion of the virus life cycle (Stubenrauch and Laimins, 1999). The genome can be divided into an early coding region, a late coding region and an 850 bp long control region (LCR) that contains *cis*-acting regulatory sequences controlling virus transcription and replication. Transcripts are synthesised from only one DNA strand and are polycistronic undergoing extensive alternative splicing and may be subject to alternative polyadenylation (Baker and Calef, 1997). The infectious life cycle is tightly linked to differentiation of the epithelial cell. Early in the virus life cycle, in cells in the lower epithelial layers, the early gene products begin to be expressed. The early proteins include E1, E2, E6 and E7. E1 is essential for virus genome replication. E2 is also important for replication but is also the virus transcription regulator (zur Hausen, 2002) and is involved in virus genome segregation during mitosis (McBride et al., 2006). E6 and E7 interact with the tumour suppressor proteins p53 and pRb respectively to inhibit apoptosis and abrogate cell cycle checkpoints to allow replication of the virus genomes in differentiated epithelial cells (Munger and Howley, 2002). E5 has been proposed to act early in infection but may also have late functions (zur Hausen, 2002). Late proteins are expressed exclusively in the upper epithelial layers. The first late protein expressed, E1^E4, is detected in the spinous and granular layers and has a number of possible functions late in infection (Doorbar et al., 1997). Expression of L1 and L2, the virus capsid proteins, is restricted to cells of the granular layer. This situation may aid immune evasion as these proteins are highly immunogenic. However, L1 and L2 RNAs can be detected in lower epithelial layers (Crum et al., 1988; Stoler et al., 1989) indicating that regulation of protein expression is post-transcriptional, likely at the level of nuclear retention and RNA decay (Koffa et al., 2000; Kennedy et al., 1991).

There have been many studies designed to map HPV transcripts. Some of these used cervical tumour cell lines where the virus life cycle has been disrupted and the late genes are not expressed (Schwarz et al., 1985; Schneider-Gädicke and Schwarz, 1986; Baker and Howley, 1987; Sherman et al., 1992). Other elegant early studies using electron microscope R-loop mapping mapped the main transcripts arising in HPV1, 6 and 11 infected tissue (Chow et al., 1987a, Palermo-Dilts et al., 1990; Chow et al., 1987b). However, RNA was prepared from whole warts. Thus, no distinction could be made between early and late mRNAs expressed in the lower and upper epithelial layers respectively. One study using *in situ* hybridisation of HPV16 precancerous lesions detected a number of late RNAs but these were not precisely mapped (Crum et al., 1988). More recently, studies of HPV31 used CIN612 9E cells that were derived from a low-grade cervical lesion induced to differentiate in semisolid medium (Meyers et al., 1992; Ruesch et al., 1998). This gives a large population of differentiated cells that can be used to study late RNAs. The first study isolated polyadenylated RNA and defined the structure of six major early region mRNAs and three late mRNAs, two encoding L1 and L2 (Hummel et al., 1992; Hummel et al., 1995). Another study examined RNAs produced in CIN612 9E organotypic raft cultures induced to differentiate by treatment with phorbol esters (Ozbun and Meyers, 1997). Nineteen different transcripts were identified that encoded at least the L1 capsid protein indicating that these represented the HPV31 late transcript population. However, RT-PCR analysis was carried out on total RNA so it is not possible to be certain that all these RNAs exist as protein-coding messenger RNAs at steady state levels in the infected cell.

Previously there have been only two studies of the transcripts synthesised by the HPV16 genome during the virus life cycle, one in KG cells (HPV16-positive vulvar intraepithelial neoplasm cell line) (Grassman et al., 1996) and one in the W12 cell line (Doorbar et al., 1990). The W12 cell line was established from cervical epithelial cells taken from a patient with a low-grade cervical lesion (Stanley et al., 1989). The cells are immortalised but not transformed and maintain up to 1000 episomal copies of the HPV16 genome in the differentiated cells (Jeon et al., 1995; Garner-Hamrick and Fisher, 2002). Most importantly, they can be induced to differentiate in monolayer culture by a number of means including culturing to high density in 1.2 mM Ca^2+^. In the W12 study, total RNA was extracted from cells grown in the absence of calcium but to high density. The differentiation status of the cells from which RNA was prepared was not determined. However, a number of RNAs encoding E6, E7, E1^E4, E5 and L1 were identified and the major splice donor and splice acceptor sites of the virus genome were mapped (Doorbar et al., 1990). In the KG study, polyadenylated RNA was isolated from raft cultures (a mix of undifferentiated and differentiated cells) of KG cells. Similar RNAs to some of those found in the W12 study were delineated and some of the splice donor/acceptor sites confirmed (Grassman et al., 1996). Neither of these studies set out to map specifically and exhaustively the HPV16 late mRNA population because the RNA populations examined were likely a mix of early and late transcripts.

Here we have used W12 cells differentiated in monolayer culture that express markers of epithelial differentiation (involucrin, filaggrin) and the virus late proteins E1^E4 and L1, and 5′RACE, together with primer extension to carry out a complete analysis of the mRNAs synthesised from the HPV16 genome. The advantage of using RACE as opposed to 5′RT-PCR is that the 5′ primer used does not define the 5′ ends of the mRNAs. In theory therefore, a full array of mRNAs with known, and undiscovered, 5′ ends can be examined. We have delineated thirteen mRNAs expressed late in the HPV16 life cycle and show two distinct transcript populations, one encoding L1 alone and the other encoding L1 and L2. Previously observed splice donor and splice acceptor sites are confirmed and other novel sites characterised. We report extensive use of the P_E4_ promoter that transcribes a monocistronic RNA encoding only L1 and frequent use of the P_late_ promoter where initiation is imprecise, covering a region of around 200 nucleotides within the E7 gene. We present evidence for a novel promoter located 157 nucleotides upstream of P_97_ in the long coding region and one located in the E5 open reading frame. Finally, we have detected novel HPV16 RNAs that may encode E6*^*E7, E1^E2C, E6*^E4, and E1^*E4, indicating that at late times of HPV infection there may be more proteins expressed than was previously considered.

## Results

### The W12 cell line differentiates efficiently and expresses late proteins

**Fig. 1:**
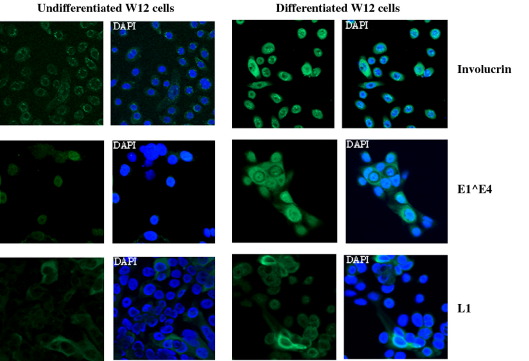
Differentiation of W12 cells populations. Immunofluorescence analysis of undifferentiated and differentiated W12 cells demonstrating expression of virus late proteins L1 and E1^E4. Nuclei are DAPI-stained. Although cells were grown at different confluence, they were plated out onto coverslips at similar density 16 h prior to fixation for microscopy.

The W12 cell line is a good model for study of the HPV16 life cycle because the cells contain many episomal copies of the HPV16 genome and can be induced to differentiate in monolayer culture (Jeon et al., 1995) to express markers of epithelial differentiation and virus late proteins (McPhillips et al., 2004). In order to ensure maintenance of the virus genomes as episomes (to abrogate integration into the host genome) and to obtain good differentiation, the cell line was used at low passage (< 17 passages). We checked routinely that the majority of genomes in the cells we cultured were episomal. On average greater than 90% of genomes remain episomal at passage 17. When the cells are cultured for up to 5 days in low concentrations of calcium at low density (< 30% confluent, few colonies), only very low levels of involucrin, a marker of suprabasal epithelial cells, are detected and expression of the first virus late protein E1^E4 is also low (Fig. 1). However, when the cells are grown for a further 5 days in 1.2 mM Ca^2+^ at over 70% confluence, between 75 and 85% of the population express involucrin indicating that most cells are differentiated. In addition, around 80% of cells express E1^E4 and around 5% of cells strongly express L1, the major capsid protein (Fig. 1). In Fig. 1, although cells labelled as undifferentiated were cultured at 30% confluence to inhibit differentiation, they were plated onto coverslips 16 h before fixing for microscopy at an equivalent density to differentiated W12 cells. The low level of involucrin staining in the undifferentiated population indicates that this procedure did not induce significant differentiation of these cells.

**Fig. 2:**
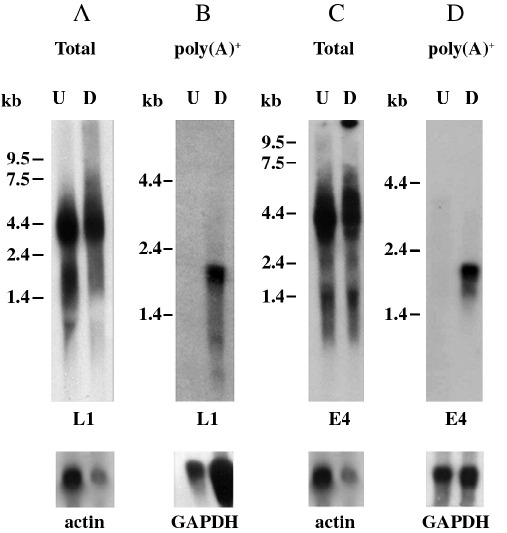
Northern blot analysis of expression of late transcripts in undifferentiated (U) and differentiated (D) W12 cells. Radiolabelled probes were either the insert from plasmid pRL1 containing a portion of the L1 gene (nts 6151–6818) or the insert from plasmid pE4 containing the entire E4 gene sequence (nts 3339–3598) (E4). Total; total cellular RNA. poly(A)^+^; polyadenylated RNA. Blots were hybridised in 50% formamide 5× SSC at 42 °C for 16 h and washed to 0.1× SSC at 65 °C. Blots were stripped and reprobed with radiolabelled actin or GAPDH sequences as loading controls.

“Undifferentiated” W12 cell RNA was prepared from populations of cells that expressed very low levels of involucrin or E1^E4, as assayed by Western blotting, while “differentiated” W12 cell RNA was prepared from cell populations that expressed high levels of involucrin and E1^E4 and showed some L1 expression. Northern blot analysis of DNase 1-digested total RNA indicated that RNAs encoding L1 were equally as abundant in undifferentiated as in differentiated W12 cells (Fig. 2A). Late transcripts detected were similar in size to those detected previously in L18 (HPV18-positive; Frattini et al., 1997) and CIN612 9E (HPV31-positive; Ozbun and Meyers, 1997; Klumpp and Laimins, 1999) cells. However, analysis of polyadenylated RNAs indicated that fully processed L1-encoding mRNAs were present only in differentiated W12 cells (Fig. 2B). A range of sizes of mRNAs from around 0.8–2 kb was detected as expected. Hybridisation of a similar Northern blot with an L2 probe failed to detect any L2-encoding mRNAs, although this probe did detect transcripts in the total RNA population (data not shown). Hybridisation of the first blot with an E1^E4 probe gave a similar pattern of transcripts (Fig. 2C), confirming that E1^E4 is encoded in a polycistronic RNA with L1. Again hybridisation to polyadenylated RNA was only in the differentiated cell population (Fig. 2D).

### 3′RACE reveals use *in vivo* of two late polyadenylation sites upon epithelial differentiation

**Fig. 3:**
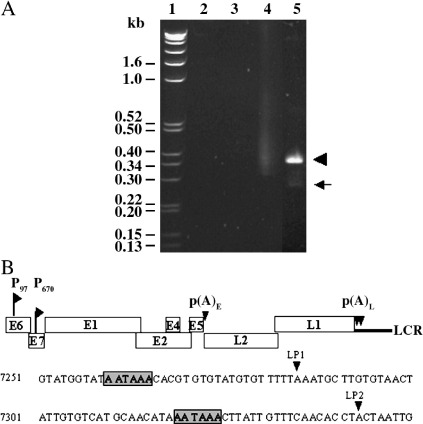
Polyadenylation of HPV16 late transcripts *in vivo*. (A) Agarose gel electrophoresis of products of 3′RACE using nested L1 gene-specific primers and cDNA reverse transcribed from polyadenylated W12 cell RNA. Track 1: DNA, marker track 2: undifferentiated RNA without reverse transcriptase, track 3: differentiated RNA without reverse transcriptase, track 4: undifferentiated RNA with reverse transcriptase, track 5: differentiated RNA with reverse transcriptase. The arrowhead indicates the major product corresponding to polyadenylation at LP2 and the arrow a minor product indicating polyadenylation at LP1. (B) Diagram of the HPV16 genome showing the approximate positions of the early and late polyadenylation sites. Below is shown the region of genome sequence from 7251 to 7350 containing the two active late polyadenylation sites. Grey boxes indicate the polyadenylation signals. Arrowheads indicate the cleavage sites.

All late transcripts are predicted to co-terminate in the late 3′UTR. Therefore, 3′RACE with nested primers located at the end of the L1 coding region (Table 1) was used to map the 3′ ends of the late mRNAs present in undifferentiated and differentiated W12 cells. No major products were amplified from cDNA reverse transcribed from polyadenylated RNA isolated from undifferentiated W12 cells (Fig. 3A, lane 4). In contrast, a major band of around 350 bp and a minor band of around 270 bp were PCR-amplified from differentiated W12 cells (Fig. 3A, lane 5). Sequence analysis of 40 cloned cDNA products from each band showed that the upper band represented use of the LP2 cleavage site at nt 7343 (AAUAAA at nt 7319) (Fig. 3B), while the smaller band represented use of the proximal LP1 cleavage site at nt 7284 (AAUAAA at nt 7260, 58 nucleotides upstream of the LP2 site). No products were obtained when reverse transcriptase was omitted from the initial reverse transcription reactions (Fig. 3A lanes 2 and 3). The results of these experiments indicate that both the LP1 and LP2 sites can be used *in vivo* in late transcripts in differentiated infected epithelial cells and that few full-length polyadenylated late transcripts are present in undifferentiated W12 cells. A polyadenylation signal in the L1 coding region active in HeLa cells transfected with an L1 expression construct was described previously (Öberg et al., 2003). 3′RACE using primers X and Y in Table 1 failed to detect use of this site in differentiated W12 cells (data not shown).

### 5′RACE detection of late transcripts synthesised in differentiated W12 cells

**Fig. 4:**
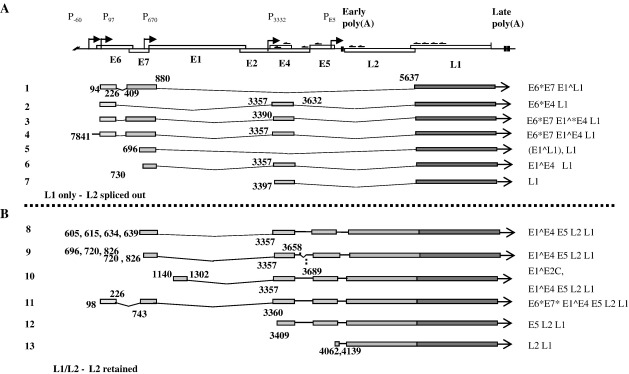
Schematic diagram of the transcripts identified by 5′RACE. (A) A diagram of the linearised virus genome. Open boxes; coding regions, arrows; promoter positions, chevrons; approximate positions of the primers used in 5′RACE. Early poly(A) and vertical bar: early polyadenylation site, late poly(A) and vertical bars: late polyadenylation sites. (B) Structures of the cDNA species identified. Grey bars: coding sequences, lines: introns spliced out. Numbers indicate the genomic positions of the 5′ ends of the cDNAs and the splice donor and acceptor sites.

cDNAs representing W12 cell late mRNAs were amplified from DNase I-treated polyadenylated RNA isolated from differentiated W12 cells using a primer located within the L1 coding region (5′ nt 6069, Table 1). C-residues were added to the 5′ end of the cDNAs using terminal deoxynucleotidyl transferase. A 5′RACE amplification primer (AP, Invitrogen) containing G- and I-residues to prime specifically from the added 5′ C-tail and one of the nested upstream L1 PCR primers (5′ nts 5968, 5776, 5690, Table 1) or L2 primers (5′ nts 4506, 4456, Table 1) was used to PCR-amplify the cDNAs. To be sure that full-length mRNAs were mapped, a genome walk was accomplished using cDNA synthesis with a series of primers located in the E5, 3′ E2 and E4 open reading frames. PCR amplification with nested 3′ primers (Table 1) and the AP 5′RACE primer was carried out. Each reaction was designed to yield populations of products that overlapped with a separate PCR reaction. The genome walk was terminated within the E4 reading frame as we were interested in late mRNAs. Transcripts encoding E1 and E2 are present late in the virus life cycle, but these terminate at the early polyadenylation site (data not shown). The strategy is diagrammed in Fig. 4A. PCR products were amplified and cloned into pGEM T-easy vector. Recombinant plasmid DNA was isolated from bacterial colonies, and at least 10 clones from each PCR reaction were sequenced. Structures of mRNAs were carefully checked by comparing sequences obtained from different PCR amplification reactions and by PCR amplification across splice junctions. Thirteen main transcript types were obtained (Fig. 4B). These could be divided into two classes, those that encoded L1 (in some cases with other open reading frames) and those that encoded both L1 and L2 (in some cases with other open reading frames). Splice donor and acceptor sites are shown in Table 2. Several mRNAs have the capacity to encode novel chimeric proteins (Table 3).

### Promoters in the E4 and E5 reading frames are used to express late transcripts

**Fig. 5:**
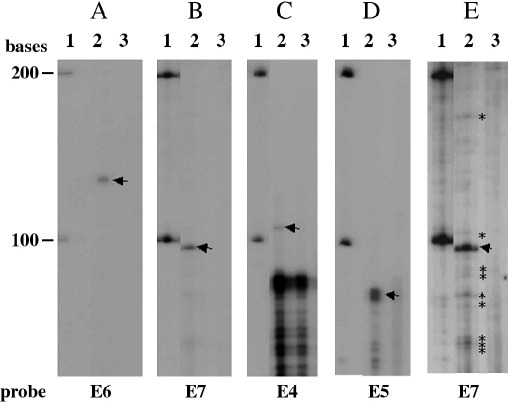
Primer extension mapping of the transcription start sites of the W12 RNAs. Tracks 1: Century RNA marker (Ambion), 2: W12 RNA, 3: HaCaT RNA. Arrows indicate the major transcript starts. Stars indicate minor transcript starts. Oligonucleotide probes used are indicated below the autoradiographs.

Two of the mRNAs identified appeared to have their 5′ ends at the start of the E4 open reading frame and one at the end of the E5 open reading frame. Promoters within E4 have been reported previously (Doorbar et al., 1990; Ozbun and Meyers, 1997). However, because cDNA synthesis could be truncated before the 5′ end of the transcript was reached, it was possible that the products we identified were representative of incomplete cDNAs. So primer extension was used to identify transcription start sites for the late mRNAs using primers that would be expected to be sited around 100 nucleotides downstream of the putative promoters predicted from the RACE analysis (Fig. 4A). Total RNA was isolated from differentiated W12 cells and from differentiated HaCaT cells as a virus-negative epithelial cell control. Each primer extension was repeated at least five times, and sequencing ladders were run on the gels, as well as the size markers shown, to accurately size the observed products. Figs. 5A and B tracks 2 show a major product of around 130 nucleotides for P_E6_ and around 97 nucleotides for P_E7_. These correspond to transcription starts at P_97_ and P_696_, in agreement with the positions of known HPV16 promoters (Smotkin and Wettstein, 1986; Doorbar et al., 1990; Grassman et al., 1996). A longer exposure of the E7 primer extension gel shows a number of subsidiary start sites over 100 nucleotides from around genome position 650 to 760 (Fig. 5E). A discrete band of 105 nucleotides, corresponding to a transcription start site at P_3392_, was observed using a primer within the E4 gene region (Fig. 5C) that corresponded to the predicted P_E4_ promoter previously observed for HPV31 (Ozbun and Meyers, 1997). Finally, two products at 86 and 84 nucleotides corresponding to a transcription start at P_4062/4064_ were observed when a primer within the E5 gene region was used (Fig. 5D), confirming the RACE data that indicated the presence of a promoter at the end of the E5 open reading frame. No primer extension products for the E6, E7, E4 and E5 primers were detected with HaCaT RNA as the starting material. Transcripts 10 appeared to start within the E1 open reading frame. As a similar transcription start had previously been identified in HPV1, it was expected that primer extension would also verify this promoter. However, no bands exclusively present in W12 cell tracks were identified. Transcripts 4 indicated the presence of a promoter in the LCR, but this could not be confirmed by primer extension indicating that transcription initiation from this promoter may be infrequent.

## Discussion

Here we report a thorough analysis of thirteen HPV16 mRNAs synthesised in differentiated W12 cells grown in monolayer. Although the monolayer population is mixed with respect to differentiation status, we estimate that between 75 and 85% of the population express involucrin, a marker of suprabasal epithelial cells, and E1^E4, the first virus late protein expressed during the HPV16 life cycle (Doorbar et al., 1997). Only around 5% of cells express the major capsid protein but by Northern blotting we have demonstrated that mRNAs encoding at least the L1 capsid protein are abundant in these cells. This means that the majority of cells within the population clearly support significant late events in the virus life cycle and that we have analysed HPV16 late mRNAs. L2 probes failed to detect L2-encoding mRNAs by Northern blot, but these were present in the late mRNA population as they were readily amplified by RACE. This may indicate that L2 mRNAs are present at a low level in the late RNA population in HPV16-infected cells perhaps reflecting the much lower requirement for L2 in capsid formation. Northern blotting also confirmed that HPV16 late gene expression is regulated post-transcriptionally because abundant RNAs encoding E4 and L1 were observed in RNA prepared from largely undifferentiated cells, but no polyadenylated E4 or L1 mRNAs were detected in this cell population. This indicates that the RNAs detected in the total RNA population are nonpolyadenylated and would therefore be unstable. Northern blotting of undifferentiated HPV31 total RNAs has also revealed long polycistronic transcripts of similar size to those observed in this study, indicating that this regulation is not specific to HPV16 (Klumpp and Laimins, 1999).

The first characterisation of mRNAs encoded by HPV16 in W12 cells was carried out on a mixed population of undifferentiated and differentiated W12 cells using defined 5′ and 3′ primers (Doorbar et al., 1990). This strategy would restrict the mRNAs found to those containing binding sites for those primers. In contrast, we have used a 3′ reverse transcriptase primer that should be present in all late mRNAs because they are predicted to end in the late 3′UTR. More importantly, we have not used a defined 5′ primer but have 5′ anchor primer-C-tailed the cDNAs synthesised and used the anchor primer as the subsequent 5′ primer. In the Doorbar study, four of the mRNAs described were clearly early region transcripts as they did not contain the late coding region. We have not yet identified the two late mRNAs they described. Ozbun and Meyers (1997) described a complex set of transcripts synthesised in organotypic raft cultures (treated with the protein kinase activator, C8:0) established from CIN612 9E cells containing episomal HPV31 genomes. The use of raft cultures ensured identification of a population of late transcripts. However, the cell population also contained undifferentiated cells and total RNA was used as the starting material for RT-PCR. This suggests that a number of the HPV31 transcripts identified may be expressed early in the life cycle and some may be unprocessed or partially processed RNAs. We used polyadenylated RNA as the substrate for cDNA synthesis in the present study so it is very likely that the transcripts we have analysed are fully processed.

We have identified a panoply of putative promoters approaching the complexity of what has been described for BPV1 (Baker and Howley, 1987). As expected, transcripts that use the well-characterised P_97_ promoter were found underlining the constitutively active nature of this promoter (Smotkin and Wettstein, 1986; Smotkin et al., 1989; Doorbar et al., 1990; Grassman et al., 1996). A series of transcription starts in the E7 open reading frame was identified. The major band detected in primer extension gave a start site of P_696_ and transcripts 5 and 9 had their 5′ ends at this position. This is 27 nucleotides downstream from the start site detected in KG cells (Grassman et al., 1996). However, the KG cell study used only primer extension to map the 5′ ends of HPV16 transcripts, whereas we have combined data from primer extension and 5′RACE, which may give a more accurate estimate. The start sites we detected covered a large region around this site, in agreement with previous studies on HPV31 (del Mar Pena and Laimins, 2001). Imprecise transcription initiation is known to occur frequently for non-TATA, non-initiator promoters (Smale, 1997), and it is likely that the E7 promoter falls into this class. There has been a report of a promoter in the E6 open reading frame at nucleotide 542 that is active in SiHa cells but is predicted to be differentiation-dependent and thus also active during the HPV16 life cycle (Glahder et al., 2003). We did not identify this transcription start site. However, if transcription initiation was imprecise on this TATA-less promoter, it may be the promoter for transcripts 8 in Fig. 4. Transcripts 10 indicated use of a promoter in the E1 open reading frame. An E1 promoter has been reported for BPV1 (Choe et al., 1989). Transcripts 10 displayed an extremely good consensus splice donor site at nucleotide 1302 with splicing to the E4 splice acceptor site, as previously noted (Doorbar et al., 1990). These mRNAs would putatively encode up to 50 amino acids from the 5′ end of E1 spliced on to the E2 open reading frame generating an E8^E2C protein and/or a novel E1^E4 product. Unfortunately, we could not prove use of the E1 promoter in the primer extension experiments. Doorbar et al. (1990) first identified a putative promoter in the E4 open reading frame. Later a similarly positioned promoter was shown to be used for production of late transcripts in HPV31 (Ozbun and Meyers, 1997). Our data indicate that this promoter, which initiates transcription at nucleotide 3397, is used relatively frequently as many cDNAs with the structure shown for transcript 7 were identified from sequencing of the products of 5′RACE. The predicted size of a transcript initiating at nucleotide 3397 with splicing at 3631 at the 3′ end of the E4 open reading frame to 5637 at the 5′ end of the L1 coding region and terminating at the major late polyadenylation site is 1858 bases. It is likely that the lower band in the E4 Northern blot of polyadenylated RNA in Fig. 2 is this mRNA. The stronger intensity upper bands of around 2 kb may be a mixture of transcripts 2–6. Comparison of primer extension and Northern blot data indicates that the P_E4_ promoter is less active than the P_late_ promoter overall. A promoter at the 3′ end of the E5 coding region has previously been described for BPV1 (Baker and Howley, 1987), HPV1 (Chow et al., 1987a, Chow et al., 1987b), HPV6b (Karlsen et al., 1996) and HPV16 and 18 (Rohlfs et al., 1991; Geisen and Kahn, 1996), but not for HPV11 or 31. Primer extension analysis confirmed the 5′RACE mapping showing transcripts that started at 4062/4064 with L2 as the first open reading frame. cDNA clones whose 5′ ends mapped to P_4062/4_ were relatively abundant and primer extension gave a band of higher intensity than that for P_97_ initiated transcripts suggesting that this initiation site was used frequently. However, because an L2 probe failed to detect mRNAs in Northern blots, possibly due to instability elements in the L2 open reading frame (Öberg et al., 2003), it is difficult to really assess how frequently this promoter is used. Finally, transcripts 4 were initiated from a promoter in the long control region. Promoters such as this have been described previously for HPV31 (Ozbun and Meyers, 1999) and BPV1 (Baker and Howley, 1987) but we did not observe a band corresponding to this initiation site in primer extension.

As expected, the W12 HPV16 late mRNAs were the products of extensive alternative splicing and also alternative polyadenylation. Most of the 5′ and 3′ splice sites we mapped agree with those mapped previously for HPV16 (Doorbar et al., 1990; Baker and Calef, 1997; Zhao et al., 2005) and correspond to those mapped for HPV31 (Ozbun and Meyers, 1997). We confirm a splice acceptor site in the E7 open reading frame at nucleotide 743, previously mapped in cervical cancer cells (Tang et al., 2006), indicating that production of this E6^E7 mRNA is not a tumour-specific splicing event. A new good consensus splice acceptor site at 3390 in the E4 open reading frame (transcript 3) was found. Use of this splice site putatively trims 11 amino acids from the 5′ end of the E4 portion of the E1^E4 protein. A number of transcripts (transcripts 9) were detected where a novel splice variant was observed indicating the presence of a very small intron at the end of the E2 open reading frame. The putative splice acceptor site is good consensus, but the splice donor site is poor (Table 2). This product could be a sequencing artefact, however, many transcripts displaying this putative intron were identified. If this splice event occurred, it would remove 11 amino acids in frame from the DNA binding/dimerisation domain of E2. There are three putative late polyadenylation sites at nucleotides 7285, 7343 and approximately 7680. Kennedy et al. (1990) reported the use *in vitro* of the first, a putative weak polyadenylation site, and the last, a putative strong polyadenylation site. We now demonstrate use *in vivo* of these two sites and confirm that site 7285 is used less frequently. It is unclear as yet whether different transcripts use one or other of these.

Table 3 describes four mRNAs that have the potential to encode novel short virus proteins. This raises the possibility that HPV16 encodes more proteins than previously considered. If so, this could extend the regulatory networks by which the virus controls host cell function. Verification of the existence of these in patient tissue is ongoing. Ultimately expression of proteins from these mRNAs requires to be demonstrated but this may prove difficult due to the expected low abundance of viral proteins in infected tissue.

Finally, these analyses begin to indicate how it is possible that the L1 and L2 proteins are efficiently translated from late transcripts. Previously, focus was on the well-characterised P_97_ and P_late_ promoters and all late mRNAs were assumed to be polycistronic. The laws of translation initiation would indicate very inefficient expression of the late proteins as they are encoded by subsequent open reading frames (Kozak, 1991). Transcripts 7 are abundant mRNAs that have L1 as the first open reading frame meaning that translation of this protein from these transcripts would be efficient. Transcripts 13 are initiated from a promoter at the 3′ end of the E5 open reading frame, and thus L2 is the first open reading frame in these mRNAs. E1^E4 and E5 are also now considered as late proteins. Transcripts 5 and 6 have E1^E4 as the first open reading frame while transcripts 12 encode E5 first. The considerable diversity of promoters used and the transcripts they initiate may allow efficient translation of proteins from mRNAs that are polycistronic in nature due to the compact organisation of the HPV genome.

## Materials and methods

### Cell culture

The 20863 (W12E) cell line is a subclone of the W12 line, an HPV16 positive cell line derived from a low-grade cervical lesion, which contains, depending on the differentiation state of the cells, up to 1000 episomal copies of the HPV16 genome (Stanley et al., 1989; Jeon et al., 1995). For monolayer culture, W12E cells were co-cultured with mitomycin C-treated J2 3T3 fibroblast feeder cells at a ratio of 1:5, seeding W12E cells at 2 × 10^5^ cells/100 mm dish (Jeon et al., 1995). The W12E line was grown for up to 5 days for undifferentiated cells (cells at around 30% confluence, no colonies), and 10 days for differentiated cells (large colonies of W12 cells), respectively. HaCaT cells, spontaneously immortalised keratinocytes (Boukamp et al., 1988), were grown in DMEM, 10% foetal calf serum and 2 mM glutamine. Cells remained undifferentiated at 30% confluence and were differentiated at around 70% confluence after growing for 6 days.

### Plasmids

pRL1 was a gift of Dr. Craig Meyers, Penn State University Medical School. It contains a *Bam*HI/*Eco*RI fragment (nts 6151–6818). pE4 was prepared by PCR amplification of the E4 gene sequence, from the W12 genome cloned into pBluescript (pEF2), using sense primer nt 3339 5′ GTCCTACATCTGTGTTTAGCAGC 3′ and antisense primer nt 3598 5′ CAGTTAATCCGTCCTTTGTGTGAGC 3′ and cloning into pGEM-Teasy vector (Promega).

### Immunofluorescence microscopy

W12 cells were grown in the presence of 3T3 feeder cells for 4 days (for undifferentiated cell populations) or 9 days (for differentiated cell populations) in 100 mm culture dishes. At day 4 or day 9, 3T3 cells were removed by trypsinisation and W12 cells at 2 × 10^5^ cells per coverslip were grown overnight on sterile 18 × 18 mm coverslips in keratinocyte growth medium (Cambrex Bioscience, UK). Cells on coverslips were washed three times with PBS and fixed in 20% sucrose, 5% formaldehyde in PBS for 10 min at room temperature. After three washes with PBS, the coverslips were permeabilised with 70% acetone, 30% methanol solution for 5 min at − 20 °C then washed three times with PBS and incubated in PBS containing 20% normal calf serum for 1 h at room temperature. Cells were incubated with primary antibodies diluted in 1% calf serum in PBS for 1 h at room temperature. Involucrin antibody (SY5, Sigma) was used at 1:1000, E1^E4 (TGV 402, (Doorbar et al., 1988)) and L1 (K1H8, Dako) at 1:50. Coverslips were washed in PBS six times before incubation for 1 h with fluorescein-labelled anti-mouse secondary antibody diluted 1:1000 in 1% (v/v) calf serum in PBS (Vector Laboratories). After washing in PBS six times, the coverslips were mounted with Vectashield mounting medium (with DAPI as a nuclear stain). Images were taken using a Zeiss LSM510 Meta confocal microscope.

### RNA preparation

Following removal of the 3T3 feeder cells by trypsinising, total RNA was extracted from W12 cells using Trizol reagent (Invitrogen) according to the manufacturer's instructions. Polyadenylated RNA was prepared by two passages of the RNA through an oligotex column using the mRNA mini kit (Qiagen) according to the manufacturer's instructions. RNA was checked for integrity by Northern blotting using a radiolabelled actin probe.

### Northern blotting

10 μg total RNA or 0.5 μg polyadenylated RNA was fractionated on a denaturing 1.2% agarose–2.2 M formaldehyde gel and Northern blotted onto Hybond-H (Amersham) as described (Sambrook et al., 1989). Blots were hybridised with [^32^P]-labelled DNA probes synthesised by random priming in 5× SSC (1× SSC is 0.15 M sodium citrate)–50% formamide at 55 °C for 16 h and washed to 0.1× SSC at 65 °C followed by autoradiography.

### 5′RACE

Polyadenylated differentiated W12 cell RNA was DNase I-treated for 1 h at 37 °C using RQ DNase I (Promega) according to the manufacturer's instructions. cDNA was synthesised using L1 RT 6069 primer (Table 1) and Superscript II reverse transcriptase (Invitrogen) at 42 °C for 1 h. Input RNA was removed by treatment with RNase H at 37 °C for 30 min. cDNA was purified using a Qiagen PCR purification kit according to the manufacturer's instructions. cDNA was 5′ end C-tailed using terminal deoxynucleotidyl transferase (Invitrogen). PCRs were carried out using 5′RACE abridged anchor primer (Invitrogen) and 3′ virus genome-specific primers (Table 1) located upstream of the primer used in the reverse transcription reaction. Subsequent PCR reactions used abridged universal amplification primer (Invitrogen) with internal 3′ primers. PCR products were fractionated on agarose gels, and bands were excised and purified using a Qiaquik gel extraction kit (Qiagen). Where products were less that 500 nucleotides, fractionation was on 6% acrylamide gels. DNA was eluted from excised bands from acrylamide gels by incubation overnight at 4 °C in 0.5 M NaCl, 1 mM EDTA pH 8.0. DNA was recovered by ethanol precipitation. Purified DNA was ligated into pGEM-Teasy vector (Promega) and transformed into XL-10 supercompetent *E. coli* cells (Stratagene). Recombinant plasmid-containing bacteria were purified and inserted DNA was sequenced.

### 3′RACE

RNA preparation, DNase I treatment and reverse transcription were exactly as for 5′RACE except oligo(dT) was used to prime first strand cDNA synthesis. 3′RACE was carried out according to the manufacturer's protocol (Invitrogen). For amplification of endogenous late transcripts in W12 cells, two gene-specific 5′ primers, outer primer 7004 and inner primer 7049 (Table 1), were used with the 3′ universal amplification primer (Invitrogen). To search for transcripts with 3′ end in the L1 open reading frame, gene-specific primers X and Y were used.

### Primer extension

DNA oligonucleotides probes were end-labelled by filling in of recessed 3′ termini. 300,000 cpm of probe was ethanol precipitated with 15 μg total RNA. The precipitated nucleic acids were resuspended in 30 μl hybridisation buffer (40 mM PIPES pH 6.4, 1 mM EDTA pH 8.0, 0.4 M NaCl, 80% formamide). The hybridisation mix was incubated at 85 °C for 10 min to denature the nucleic acids. Hybridisation was carried out at the desired annealing temperature overnight. Following overnight incubation, the hybrids were ethanol precipitated and resuspended in 20 μl reverse transcriptase buffer (50 mM Tris–HCl pH 7.6, 60 mM KCl, 10 mM MgCl_2_, 1 mM dNTPs, 1 mM DTT, 1 unit RNasin (Promega)). Fifty units of Superscript II (Invitrogen) was added and the reaction incubated for 1 h at 42 °C. The template was removed by addition of EDTA to 25 mM and DNase-free RNase to 0.25 μg/ml and incubation at 37 °C for 30 min. The mixture was then phenol/chloroform extracted and ethanol precipitated. The washed pellet was dissolved in 6 μl formamide loading buffer and the products denatured by heating at 95 °C for 5 min and resolved by electrophoresis through a 6% denaturing polyacrylamide/urea gel.

## Figures and Tables

**Table 1:** Oligonucleotides used in this study

*5′RACE*	
L1 6069 (RT)	CTCTATTATCCACACCTGCAT
LI 5968	TGACCACGACCTACCTCAACACCTACACA
L1 5776	TGTCCAACTGCAAGTAGTCTGGATGTTCCT
LI 5690	TACTGGGACAGGAGGCAAGTAGACAGTGGCCTCAC
L2 4506	GGTCTTACAGGAGCAAGTGTATCTGTAGCT
L2 4456	TGGAATATACCCAGTGCGTCCGCCTGTA
E5 3890	GCACGCCAGTAATGTTGTGGATGC
E2 3845	GACATAAATCCAGTAGACACTGTAAT
E4 3596	CAGTTAATCCGTCCTTTGTGTGAGCT
E4 3451	CTTCGGTGCCCAAGGCGACGGCTTTGGTAT

*3′RACE*	
Outer primer 7004	CTGCAGACCTAGATCAGTTTC
Inner primer 7047	CAAGCAGGATTGAAGGCC
Primer X 5746	CCAGACTACTTGCAGTTGGAC
Primer Y 5948	TGTTGAGGTAGGTCGTGGTCAGC

*Primer extension*	
E6 222	CGTCGCAGTAACTGTTGCTTGCAGTACAC
E7 766	CGCACAACCGAAGCGTAGAGTCACAC
E1 979	CGTTCTCGTCATCTGATATAGCATCCC
E4 3412	CGGGGTGGTTGGCCAAGTGCTGCC
E5 4126	GTAACAATTACATTATGTACATATACATTATG

**Table 2:**
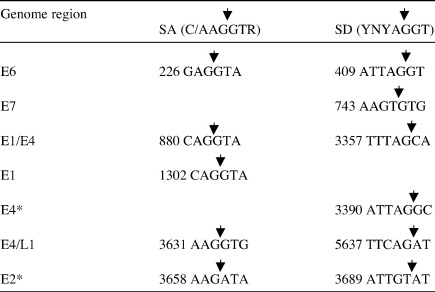
Splice donor and acceptor sites used in mRNAs synthesised in differentiated W12 cells

Consensus splice acceptor and donor sites are indicated at the top in brackets. * Novel splice sites identified in this study. Arrowheads indicate splice junctions.

**Table 3:** Putative novel proteins encoded by the W12 HPV16 genome

Transcript no.	Coordinates	Putative protein	Size (aa)
2	P_97_ promoter	E6*^E4	48
SA: 226 SD: 3357
3	P_97_ promoter	E1^E4*	81
SA: 880 SD: 3390
10	Promoter unmapped	E1^E2C	177
SA: 1302 SD: 3357
11	P_97_ promoter	E6*^E7*	53
SA: 226 SD: 743
